# Candida auris Response in a Tennessee Dialysis Facility, 2023

**DOI:** 10.1017/ash.2024.112

**Published:** 2024-09-16

**Authors:** Alex Kurutz, Joshua Key, Autumn Edwards, Simone Godwin

**Affiliations:** Tennessee Department of Health

## Abstract

**Background:** Candida auris, a multi-drug resistant fungal pathogen, was introduced to Tennessee in 2021. There are limited studies on the spread of C. auris in highly specialized care settings including outpatient dialysis facilities. Facilities are concerned that C. auris transmission is difficult to prevent in this setting due to patient vulnerability, treatment frequency and length, and isolation challenges. As a result, these facilities may reject patients based on their positive colonization status. In 2023, the Tennessee Department of Health (TDH) conducted two containment-driven colonization screenings in response to a colonized patient receiving dialysis treatment for one month without their status being known to the facility. **Methods:** An initial point prevalence survey (PPS) was conducted to assess for ongoing transmission among dialysis patients. Patient screening was prioritized for the cohort of patients who received dialysis at the same time as the index patient (Cohort A). The screening was broadened to include patients dialyzed directly before Cohort A (Cohort B) by request of the Cohort B patients. A second PPS was conducted 7 weeks later, targeting the same cohorts. Specimens were collected through supervised patient self-collection of a skin swab from the axilla and groin. Flocked Eswabs were used for collection and transferred in Amies transport media to the Tennessee State Public Health Lab. The presence of C. auris was detected via Polymerase Chain Reaction (PCR). **Results:** Twenty-three patients (12 from Cohort A; 11 from Cohort B) were screened in the first PPS. One patient from Cohort A tested positive. This colonized patient was determined to be a known C. auris case first detected four months prior, but the patient’s status was never communicated to the dialysis facility from the discharging acute care facility. Eleven patients, excluding the previously identified positives, participated (9 from Cohort A; 2 from Cohort B) in the second PPS; no positives were identified. **Discussion:** The index patient and an additional patient identified by the PPS both received dialysis at this facility for up to 4 months without facility knowledge. These results suggest that the standard infection control practices at this dialysis facility were adequate to prevent the transmission of C. auris among dialysis patients on multiple shifts. Additionally, patient self-collection identified a known C. auris patient. Future TDH work includes further evaluating the risk of C. auris transmission and developing targeted infection prevention and control practices for the outpatient dialysis setting.

